# Uptake patterns and predictors of colorectal cancer screening among adults resident in Spain: A population-based study from 2017 to 2020

**DOI:** 10.3389/fpubh.2023.1151225

**Published:** 2023-03-13

**Authors:** Silvia Portero de la Cruz, Jesús Cebrino

**Affiliations:** ^1^Research Group GC12 Clinical and Epidemiological Research in Primary Care, Instituto Maimónides de Investigación Biomédica de Córdoba (IMIBIC), Hospital Universitario Reina Sofía, Córdoba, Spain; ^2^Department of Preventive Medicine and Public Health, University of Seville, Seville, Spain

**Keywords:** colorectal neoplasms, guideline adherence, health services, healthcare disparities, mass screening

## Abstract

**Background:**

Although population screening has improved the early diagnosis of colorectal cancer (CRC), most cases are diagnosed in symptomatic patients. This study aimed to estimate the prevalence and evolution over time of uptake patterns to fecal immunochemical test (FIT) as a screening test for CRC among individuals aged 50–69 in Spain, and to determine the predictive factors for uptake patterns to this type of screening based on sociodemographic, health, and lifestyle characteristics.

**Methods:**

A cross-sectional study with 14,163 individuals from the 2017 Spanish National Health Survey and the 2020 European Health Survey was performed, including as the main variable uptake pattern to FIT screening within the last 2 years, with which we analyzed sociodemographic factors, health status and lifestyle habits.

**Results:**

38.01% of participants had undergone FIT in the previous 2 years, and from 2017 to 2020, a significant increase in the uptake rate for CRC was observed (2017: 32.35%, 2020: 43.92%, *p* < 0.001). The positive predictors to FIT uptake pattern included factors such as being between 57 and 69 years, having a higher educational level or a higher social class, having at least one chronic illness, frequent contact with the primary care physician, alcohol consumption and physical activity, while immigration and smoking habit were negative predictors.

**Conclusion:**

In Spain, although the evolution of FIT uptake pattern over time is positive, the prevalence of FIT uptake is still low (38.01%), not reaching the level considered as acceptable in the European guidelines. Moreover, there are disparities in CRC screening uptake among individuals.

## 1. Introduction

Colorectal cancer (CRC) is the third biggest cause of cancer-related mortality in both men and women globally (1). In 2020, 40,441 new cases of CRC were recorded in Spain, accounting for up to 14.3% of all new cancer diagnoses (2). By 2040, the incidence of CRC is expected to rise by 27.81%, probably due to demographic changes (3). Moreover, CRC was the second-leading cause of mortality after lung cancer in 2020, with 16,470 deaths (4).

In Spain, the 5-year survival rate for CRC is 63.3% (5), and although changes in CRC risk factors typically result in a decrease in incidence and death rates, this occurs over a long rather than a short time frame, which means that a considerable period of time is needed to observe and analyze how changes in CRC risk factors prevalence impact incidence and mortality rates (6). As a result, different early detection strategies have been developed, with the assumption that early treatment of the diagnosed lesions may improve the prognosis of this tumor, thus reducing its gravity or lowering mortality (7). In fact, early detection with regular screening beginning at the age of 50 is successful in enhancing survival from CRC (the 5-year survival rate in screen-detected CRC is about 93%) (8), although high screening participation rates are required to achieve this.

Following the recommendations of the European Screening Guidelines for CRC (9) and the National Health System's cancer plan (10), a CRC screening program was gradually introduced in Spain beginning in 2000. This screening program is aimed at people aged 50–69 years and is conducted using a personal invitation letter every 2 years, which they are asked to perform a fecal immunochemical test (FIT), followed by endoscopic procedures to confirm the positive test (11). In recent years, the immunohistochemical fecal test has emerged as the primary choice for screening, since it is more sensitive and has a higher diagnostic accuracy for CRC than the traditional guaiac-based fecal occult blood test (12).

CRC screening in Spain is well-organized (11) and does a better job than opportunistic screenings in terms of usage, reducing not just cancer mortality but also inequalities in access to and uptake of CRC screening, as well as being more cost-effective and enabling a larger number of individuals to be reached (13). Nevertheless, previous research performed in Spain has shown that uptake pattern for CRC screening is below 32% (14), which is lower than in other developed nations with comparable programs, such as England, Finland, Ireland, or Denmark (15). Moreover, the FIT uptake pattern rate recorded in Spain is much lower than the acceptable rate of uptake in European standards of over 45%, and ideally 65% to produce a significant benefit (9).

Given these considerations, added to the fact that the vast majority of CRC cases are still discovered in symptomatic subjects (16), it is critical to achieve the greatest possible FIT uptake patterns rate and increase the effectiveness of CRC detection programs, with a specific emphasis on the factors that impact uptake. For this reason, the aims of this study were to estimate the prevalence and evolution over time of uptake patterns to FIT as a screening test for CRC among individuals aged 50–69 in Spain, and to determine the predictive factors for uptake patterns to this screening test based on sociodemographic, health, and lifestyle characteristics.

## 2. Material and methods

A cross-sectional study was conducted, utilizing data from the Spanish National Health Survey 2017 (SNHS) (17) and European Health Survey in Spain 2020 (EHSS) (18). The National Statistics Institute performed both surveys under the auspices of the Spanish Ministry of Health and Social Affairs, using the same methods. The SNHS 2017 data collection period was from October 2016 to October 2017, while the EHSS 2020 was collected between July 2019 and July 2020.

Individuals ≥ 15 years old were chosen using probabilistic multistage sampling, with the first-final units (individuals) chosen using random routes and sex-based and age-based quotas. Trained interviewers visited randomly-chosen homes and asked the residents to participate in the survey. Computer-assisted personal interviews were also used to obtain data. Additional details about the survey methodology may be found elsewhere (17, 18).

For study reasons, in accordance with the age guideline for FIT screening, we selected individuals aged 50–69 years (11). The total sample included 15,240 records: 7,687 from SNHS 2017 and 7,553 from EHSS 2020. Despite having identical characteristics to the others, 1,077 subjects (7.07%) were subsequently removed from the total sample due to their refusal to complete the surveys (SNHS 2017: *n* = 453; EHIS 2020: *n* = 624). Finally, the study sample consisted of 14,163 participants (7,234 from SNHS 2017 and 6,929 from EHSS 2020).

The current study incorporates the self-reported responses from these questionnaires. To generate all the variables in our study accurately, we used identical questions in both surveys. Uptake pattern to FIT-based CRC screening was established as the dependent variable. The participants responded two questions: (i) “Have you ever had a fecal occult blood test?” (“Yes,” “No”) and (ii) “How long has it been since you last underwent a fecal occult blood test?” (In the last 12 months, “More than 1, but <2 years ago,” “More than 2, but <3 years ago,” and “More than 3, but <5 years ago,” and “More than 5 years ago”). According to their answers, participants were classified as:

- Never-users: those who answered “No” the first question, therefore they had never undergone a FIT.
- Uptakers: participants who answered affirmatively to the first question and answered in the second question: “In the last 12 months” and “More than 1, but <2 years ago,” in other words, individuals who had taken a FIT during the previous 2 years.
- Under-users: subjects who answered affirmatively to the first question and responded in the second question: “More than 2, but <3 years ago” or “More than 3, but <5 years ago” or “More than 5 years ago,” meaning subjects who reported that they had undergone FIT more than 2 years.
- Non-uptakers: individuals defined as “never-user” or “under-user.”

The independent variables listed below were included:

- Sociodemographic factors such as gender (men/women), age group (50–56/57–63/64–69), level of education (without studies/primary/secondary/university), marital status (single/married/widowed/separated-divorced), social class (upper/middle/lower) (19), residential location (rural/urban), and nationality (Spanish/foreign).
- Variables related to health status, such as number of chronic diseases (0/1/ ≥2), presence of physician-diagnosed mental illness (yes/no), self-perception of health status in the last 12 months (very good/good/average/bad/very bad), insurance status (public/private), visits to the primary care physician in the last month (yes/no), and visits to a medical specialist in the last month (yes/no).
- Lifestyle habits, including body mass index (underweight/normal weight/overweight/obesity) (20) current smoking habit (yes/no), alcohol consumption in the last year (yes/no), and free time physical exercise (yes/no).

Permission from an ethics committee is not required under Spanish law, because the database was obtained from the website of Spanish Ministry of Health, which is accessible to the public.

The frequencies and percentages were used to provide the descriptive analysis of qualitative variables. For comparisons, we used chi-squared test. A binary logistic regression was also performed to determine the predictors of the FIT uptake pattern. We calculated crude and adjusted odds ratio (OR), as well as their 95% confidence intervals. We utilized the Wald statistic, in which the variables with *p* < 0.15 were removed from the model one by one. The Hosmer–Lemeshow test was used to assess the quality of fit, and measure the goodness of fit, and we examined the adjusted coefficient of determination (*R*^2^), the F statistic and the normality of the residues. All the contrasts of hypotheses were bilateral, and statistical significance was set at *p* < 0.05. The statistical analysis was carried out using the statistical program IBM SPSS Statistics version 25.0, which was licensed to the University of Córdoba (Spain).

## 3. Results

The sample was composed of 14,163 records of individuals aged 50–69 years. Among the participants, 50.77% were women with a mean age of 59.08 ± 5.69 years. The highest values of compliance with FIT were observed in people with a university education (39.92%), widowed (40.71%), belonging to the upper class (41.93%), living in rural residences (39.43%), Spanish nationality (38.69%), having, at least, two chronic illnesses (42.84%), suffering from a mental illness (42.11%), having a very poor self-perceived health status (43.07%), having public health insurance (38.33%), visiting a primary care physician (43.39%) or a specialist physician (46.79%) in the 4 weeks preceding survey completion, being a non-smoker (40.19%), consuming alcohol in the last year (39.41%) and doing free time physical activity (40.42%; Table 1).

**Table 1:** Uptake of fecal immunochemical test according to sociodemographic, health and lifestyle characteristics (*n* = 14,163).

**Variables**	**Uptake of FIT**			
	**Total**	**Yes**	**No**	***p*** **-value**
	***n*** = **14,163 (%)**	***n*** = **5,383 (%)**	***n*** = **8,780 (%)**	
**Gender**				
Man	6,972 (49.23)	2,628 (37.69)	4,344 (62.31)	0.45
Woman	7,191 (50.77)	2,755 (38.31)	4,436 (61.69)	
**Age group**				
50–56 years old	5,311 (37.50)	1,586 (29.86)	3,725 (70.14)	<0.001
57–63 years old	5,025 (35.48)	2,052 (40.84)	2,973 (59.16)	
64–69 years old	3,827 (27.02)	1,745 (45.60)	2,082 (54.40)	
**Level of education**				
Without studies	87 (0.61)	16 (18.39)	71 (81.61)	<0.001
Primary	3,789 (26.75)	1,299 (34.28)	2,490 (65.72)	
Secondary	7,662 (54.10)	3,020 (39.42)	4,642 (60.58)	
University	2,625 (18.54)	1,048 (39.92)	1,577 (60.08)	
**Marital status**				
Single	2,112 (14.91)	702 (33.24)	1,410 (66.76)	<0.001
Married	9,130 (64.46)	3,561 (39.00)	5,569 (61.00)	
Widowed	1,066 (7.53)	434 (40.71)	632 (59.29)	
Separated or divorced	1,855 (13.10)	686 (36.98)	1,169 (63.02)	
**Social class**				
Lower	6,592 (46.54)	2,284 (34.65)	4,308 (65.35)	<0.001
Middle	4,914 (34.70)	1,985 (40.39)	2,929 (59.61)	
Upper	2,657 (18.76)	1,114 (41.93)	1,543 (58.07)	
**Residential location**				
Urban	6,428 (45.39)	2,333 (36.29)	4,095 (63.71)	<0.001
Rural	7,735 (54.61)	3,050 (39.43)	4,685 (60.57)	
**Nationality**				
Spanish	13,383 (94.49)	5,178 (38.69)	8,205 (61.31)	<0.001
Foreigner	780 (5.51)	205 (26.28)	575 (73.72)	
**Number of chronic conditions**				
0	2,954 (20.86)	787 (26.64)	2,167 (73.36)	<0.001
1	2,629 (18.56)	920 (34.99)	1,709 (65.01)	
≥2	8,580 (60.58)	3,676 (42.84)	4,904 (57.16)	
**Presence of physician-diagnosed mental illness**				
No	11,881 (83.89)	4,422 (37.22)	7,459 (62.78)	<0.001
Yes	2,282 (16.11)	961 (42.11)	1,321 (57.89)	
**Self-perceived health status**				
Very good	1,906 (13.46)	658 (34.52)	1,248 (65.48)	<0.001
Good	7,566 (53.42)	2,787 (36.84)	4,779 (63.16)	
Fair	3,427 (24.20)	1,399 (40.82)	2,028 (59.18)	
Poor	990 (6.99)	421 (42.53)	569 (57.47)	
Very poor	274 (1.93)	118 (43.07)	156 (56.93)	
**Insurance status**				
Public	13,424 (94.78)	5,145 (38.33)	8,279 (61.67)	< 0.01
Private	739 (5.22)	238 (32.21)	501 (67.79)	
**Visits to the primary care physician in the previous 4 weeks**				
No^*^	10,194 (71.98)	3,661 (35.91)	6,533 (64.09)	<0.001
Yes	3,969 (28.02)	1,722 (43.39)	2,247 (56.61)	
**Visits to the specialist physician in the previous 4 weeks**				
No^*^	12,291 (86.78)	4,507 (36.67)	7,784 (63.33)	<0.001
Yes	1,872 (13.22)	876 (46.79)	996 (53.21)	
**Body Mass Index**				
Normal weight	4,975 (35.13)	1,865 (37.49)	3,110 (62.51)	0.19
Underweight	156 (1.10)	71 (45.51)	85 (54.49)	
Overweight	6,139 (43.35)	2,357 (38.39)	3,782 (61.61)	
Obesity	2,893 (20.42)	1,090 (37.68)	1,803 (62.32)	
**Current smoking habit**				
No^†^	10,491 (74.07)	4,216 (40.19)	6,275 (59.81)	<0.001
Yes	3,672 (25.93)	1,167 (31.78)	2,505 (68.22)	
**Alcohol intake in the last year**				
No^‡^	4,214 (29.75)	1,462 (34.69)	2,752 (65.31)	<0.001
Yes	9,949 (70.25)	3,921 (39.41)	6,028 (60.59)	
**Free time physical exercise**				
No^**^	4,792 (33.83)	1,595 (33.28)	3,197 (66.72)	<0.001
Yes	9,371 (66.17)	3,788 (40.42)	5,583 (59.58)	

FIT, Fecal immunochemical test.

* No: between 4 weeks and 12/12 months or more ago/never.

† No: I don't currently smoke, but I have in the past/I don't smoke and have never smoked on a regular basis.

‡ No: I have not consumed alcohol in the last 12 months.

** No: I do not practice any physical activity in my free time.

**Figure 1:**
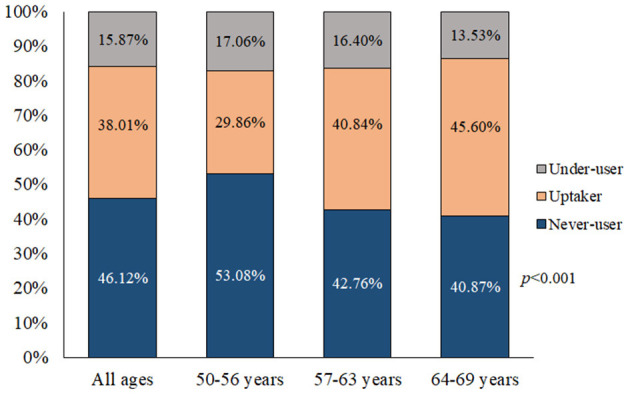
Prevalence of fecal occult blood test use by age group (*n* = 14,163).

The overall percentage of participants who had never undergone FIT was 46.12%, a figure which had decreased from 2017 (50.19%) to 2020 (41.87%; *p* < 0.001). The overall prevalence of FIT under-users was 15.87%, which decreased over the years studied (2017: 17.46%, 2020: 14.21%, *p* < 0.001). The overall percentage of FIT uptakers was 38.01%, which increased over time (2017: 32.35%, 2020: 43.92%, *p* < 0.001). Moreover, the prevalence of FIT uptakers was highest at age 64–69 (45.60%, *p* < 0.001; Figure 1).

Table 2 shows the FIT uptake in the different autonomous communities during the study period (2017–2020). Navarra (59.86%), Cataluña (52.26%), and País Vasco (51.41%) were the autonomous communities with the highest percentage of FIT, while those with the lowest proportions were Andalucía (16.97%), Asturias (19.16%), and Extremadura (22.58%; *p* < 0.001).

**Table 2:** Uptake of fecal immunochemical test of Spanish people in the period 2017–2020 in the different regions of Spain.

**Autonomous community**	**Year of CRC screening implementation**	**Uptakers of FIT**	***p*-value**
		***n* = 5,383 (38.01%)**	
Andalucía	2014	325 (16.97)	
Aragón	2013	94 (50.27)	
Asturias	2014	132 (19.16)	
Baleares	2015	200 (45.25)	
Canarias	2009	251 (34.76)	
Cantabria	2008	252 (41.11)	
Castilla y León	2010	351 (45.23)	
Castilla la Mancha	2015	425 (63.15)	
Cataluña	2000	763 (52.26)	
Comunidad Valenciana	2005	591 (48.28)	<0.001
Extremadura	2017	168 (22.58)	
Galicia	2013	277 (33.54)	
Madrid	2017	419 (31.72)	
Murcia	2005	169 (27.35)	
Navarra	2013	343 (59.86)	
País Vasco	2009	401 (51.41)	
La Rioja	2010	142 (32.35)	
Ceuta	2017	61 (32.24)	
Melilla	2017	19 (30.16)	

FIT, Fecal immunochemical test.

In general, the FIT uptake rate increased from 2017 to 2020 in each group of variables analyzed, except for people without studies and those with a self-perceived very poor health status, in which FIT uptake rate did not vary over time (Table 3).

**Table 3:** Distribution of uptakers of fecal occult blood testing, according to sociodemographic, health and lifestyle variables from 2017 to 2020 (*n* = 5,383).

	**Uptakers of FIT**		
**Variables**	**2017**	**2020**	***p*** **-value**
	***n*** = **2,340 (%)**	***n*** = **3,043 (%)**	
**Gender**			
Man	1,151 (32.72)	1,477 (42.76)	<0.001
Woman	1,189 (32.00)	1,566 (45.06)	<0.001
**Age group**			
50-56 years old	701 (25.24)	885 (34.93)	<0.001
57-63 years old	876 (34.93)	1,176 (46.72)	<0.001
64-69 years old	763 (39.15)	982 (52.29)	<0.001
**Level of education**			
Without studies	11 (17.46)	5 (20.83)	0.72
Primary	644 (30.39)	655 (39.22)	<0.001
Secondary	1,272 (33.27)	1,748 (45.53)	<0.001
University	413 (33.60)	635 (45.49)	<0.001
**Marital status**			
Single	296 (30.08)	406 (35.99)	< 0.01
Married	1,595 (32.93)	1,966 (45.86)	<0.001
Widowed	199 (34.97)	235 (47.28)	<0.001
Separated or divorced	250 (29.83)	436 (42.87)	<0.001
**Social class**			
Lower	1,027 (30.05)	1,257 (39.60)	<0.001
Middle	883 (34.72)	1,102 (46.48)	<0.001
Upper	430 (33.78)	684 (49.42)	<0.001
**Residential location**			
Urban	1,114 (31.27)	1,219 (42.53)	<0.001
Rural	1,226 (33.39)	1,824 (44.89)	<0.001
**Nationality**			
Spanish	2,293 (33.00)	2,885 (44.83)	<0.001
Foreigner	47 (16.43)	158 (31.98)	<0.001
**Number of chronic conditions**			
0	295 (22.50)	492 (29.95)	<0.001
1	358 (28.44)	562 (41.02)	<0.001
≥2	1,687 (36.17)	1,989 (50.79)	<0.001
**Presence of physician-diagnosed mental illness**			
No	1,876 (31.66)	2,546 (42.75)	<0.001
Yes	464 (35.47)	497 (51.03)	<0.001
**Self-perceived health status**			
Very good	242 (27.75)	416 (40.23)	<0.001
Good	1,152 (30.78)	1,635 (42.77)	<0.001
Fair	660 (34.90)	739 (48.11)	<0.001
Poor	222 (39.22)	199 (46.93)	0.02
Very poor	64 (39.51)	54 (48.21)	0.15
**Insurance status**			
Public	2,245 (32.66)	2,900 (44.27)	<0.001
Private	95 (26.32)	143 (37.83)	<0.001
**Visits to the primary care physician in the previous 4 weeks**			
No^*^	1,444 (29.68)	2,217 (41.61)	<0.001
Yes	896 (37.84)	826 (51.59)	<0.001
**Visits to the specialist physician in the previous 4 weeks**			
No^**^	1,856 (30.51)	2,651 (42.71)	<0.001
Yes	484 (42.09)	392 (54.29)	<0.001
**Body Mass Index**			
Normal weight	772 (31.05)	1,093 (43.91)	<0.001
Underweight	38 (48.10)	33 (42.86)	0.02
Overweight	1,019 (32.58)	1,338 (44.44)	<0.001
Obesity	511 (33.16)	579 (42.83)	<0.001
**Current smoking habit**			
No^†^	1,829 (34.29)	2,387 (46.29)	<0.001
Yes	511 (26.89)	656 (37.02)	<0.001
**Alcohol intake in the last year**			
No^‡^	697 (32.27)	765 (37.24)	< 0.01
Yes	1,643 (32.38)	2,278 (46.73)	<0.001
**Free time physical exercise**			
No^**^	715 (28.99)	880 (37.83)	<0.001
Yes	1,625 (34.08)	2,163 (46.99)	<0.001

FIT, Fecal immunochemical test.

* No: between 4 weeks and 12/12 months or more ago/never.

† No: I don't currently smoke, but I have in the past/I don't smoke and have never smoked on a regular basis.

‡ No: I have not consumed alcohol in the last 12 months.

** No: I do not practice any physical activity in my free time.

Table 4 shows the factors associated with FIT uptake among subjects aged 50–69. Compared to individuals aged 50–56, participants aged 57–63 or 64–69 were more likely to show a higher FIT uptake. While more educated subjects were more likely to have taken a FIT within the 2 previous years, the opposite was found for foreign people. In addition, a trend toward a higher probability of FIT uptake with increasing social class was observed. A similar result was observed for individuals who had been diagnosed with a chronic disease. Furthermore, participants who had had an appointment with the primary care physician in the last 4 weeks increased their probability of compliance with FIT. Finally, the individuals who had consumed alcohol during the previous year and had performed physical activity during their leisure time were more likely to have been screened, while the opposite was observed for smokers.

**Table 4:** Determinants of fecal immunochemical test among subjects aged 50–69 residing in Spain (*n* = 14,163).

**Variables**	**OR (CI 95%)**	ORa^*^(CI 95%)	***p*-value**
**Gender**			
Man	Reference		
Woman	0.97 (0.91–1.04)		
**Age group**			
50–56 years old	Reference	Reference	
57–63 years old	1.62 (1.49–1.76)	1.53 (1.41–1.67)	<0.001
64–69 years old	1.97 (1.81–2.15)	1.83 (1.66–2.01)	<0.001
**Level of education**			
Without studies	Reference	Reference	
Primary	2.32 (1.34–4.00)	1.96 (1.12–3.42)	0.02
Secondary	2.89 (1.68–4.98)	2.56 (1.45–4.51)	<0.001
University	2.95 (1.71–5.10)	2.81 (1.61–4.91)	< 0.01
**Marital status**			
Single	Reference		
Married	1.28 (1.16–1.42)		
Widowed	1.38 (1.19–1.61)		
Separated or divorced	1.18 (0.13–1.34)		
**Social class**			
Lower	Reference	Reference	
Middle	1.28 (1.18–1.38)	1.17 (1.08–1.26)	<0.001
Upper	1.36 (1.24–1.49)	1.25 (1.11–1.41)	<0.001
**Residential location**			
Urban	Reference		
Rural	1.14 (1.07–1.22)		
**Nationality**			
Spanish	Reference	Reference	
Foreigner	0.57 (0.48–0.67)	0.68 (0.57–0.80)	<0.001
**Number of chronic conditions**			
0	Reference	Reference	
1	1.48 (1.32–1.66)	1.38 (1.22–1.55)	<0.001
≥2	2.06 (1.88–2.26)	1.86 (1.69–2.05)	<0.001
**Presence of physician-diagnosed mental illness**			
No	Reference		
Yes	1.23 (1.12–1.34)		
**Self-perceived health status**			
Very good	Reference		
Good	1.11 (0.99–1.23)		
Fair	1.31 (1.17–1.47)		
Poor	1.40 (1.20–1.64)		
Very poor	1.44 (1.11–1.86)		
**Insurance status**			
Public	Reference		
Private	0.76 (0.65–0.90)		
**Visits to the primary care physician in the previous 4 weeks**			
No^*^	Reference	Reference	
Yes	1.37 (1.27–1.47)	1.23 (1.13–1.33)	<0.001
**Visits to the specialist physician in the previous 4 weeks**			
No^*^	Reference		
Yes	1.52 (1.38–1.68)		
**Body Mass Index**			
Normal weight	Reference		
Underweight	1.39 (1.01–1.92)		
Overweight	1.04 (0.96–1.12)		
Obesity	1.01 (0.92–1.11)		
**Current smoking habit**			
No**^†^**	Reference		
Yes	0.69 (0.64–0.75)	0.76 (0.70–0.83)	<0.001
**Alcohol intake in the last year**			
No^‡^	Reference		
Yes	1.22 (1.14–1.32)	1.24 (1.15–1.35)	<0.001
**Free time physical exercise**			
No^**^	Reference		
Yes	1.36 (1.26–1.46)	1.24 (1.15–1.34)	<0.001

^*^No: between 4 weeks and 12/12 months or more ago/never.

^†^No: I don't currently smoke, but I have in the past/I don't smoke and have never smoked on a regular basis.

^‡^No: I have not consumed alcohol in the last 12 months.

^**^No: I do not practice any physical activity in my free time.

OR, odds ratio.^*^ORa, odds ratio adjusted for all socio-demographic characteristics, health-related status and lifestyle behaviors; CI 95%, 95% Confidence Interval.

Hosmer-Lemeshow test χ^2^ = 11.77, p = 0.16; Nagelkerke's R^2^ Square = 0.41; *p*-value < 0.001.

## 4. Discussion

The present study used national representative surveys to analyze FIT uptake in Spain from 2017 to 2020 and to identify the variables associated with screening compliance among 14,163 individuals aged 50 to 69.

According to our findings, almost half the Spanish population had never taken a FIT, despite being in the age range suitable for CRC screening. Furthermore, a part of the screened Spanish population does not adhere to the guidelines for test intervals. The Health Ministry of Spain set an objective in 2014 of a 100% adherence rate to FIT in the 50–69 year-old population residing in Spain by 2025 (21); nevertheless, given our findings, this seems implausible.

The uptake pattern rate to FIT found in the current study was 38.01%, increasing from 32.35% in 2017 to 43.92% in 2020, demonstrating a substantial 11.57% rise. It is difficult to assess CRC screening uptake patterns across European countries since preventative screening programs differ in terms of updating data, target age groups, screening intervals and the principal test utilized in each country (22). Nevertheless, the screening rates in other European countries with analogous programs are significantly >38.01% found in the current study, for example, France (51%) or Slovenia (56%) (13). The increase in uptake observed in the current study between 2017 and 2020 might be related to the adoption of the FIT over the guaiac-fecal occult blood test in most screening programmes in Spain, which is related to higher participation among people invited in organized screening settings (23).

Despite the increase observed in Spain, and considering that the last 5 months of the 2020 data collection were conducted during the COVID-19 pandemic, restrictions in screening activities may have influenced the probability of undergoing screening during that period, limiting the percentage of uptakers and delaying CRC diagnosis. Moreover, the impact of the screening programmes cancellation could be longer than the period they were closed, because program's restart was progressive and many people could have decided not to participate in the programmes to prevent unnecesary virus exposure (24). Some researchers have examined the effect of the cancellation of these CRC screening programmes on CRC. In that sense, a recent study showed that delaying CRC screening by 4–6 months would rise the number of advanced CRC cases and even mortality if delayed for more than 12 months (25). It is critical that health authorities officials ensure that the general population recognizes how essential these programmes are. On the other hand, it is important to highlight that the low level of FIT adoption in Spain could be influenced by the uneven implementation of the CRC screening program (10), owing to the fact that each region has a separate public health system overseen by its own regional government, despite the fact that Spain's health system is public. This unequal implementation was due to each region having one public health system that is managed by a different regional government, even though the health system in Spain is public.

In Spain, Catalonia was the first autonomous community to carry out screening programs with a pilot study in 2000 (26) and, in our study, was the region with the highest percentage of FIT (52.26%) during 2017–2020. This percentage of FIT contrast with those obtained in Andalucía (16.97%), Asturias (19.16%), or Extremadura (22.58%), where CRC screening was more recently implemented, which is generating a growing demand for opportunistic CRC screening (27). The invitation to opportunistic screening is sporadic and is established by individual initiative or by general practitioners or specialized physicians. Its benefit in terms of morbidity and mortality has not been proven, there is no guarantee of quality control, and it is less equitable and likely less efficient (27).

FIT screening compliance varies not only depending on the country, but also according to sociodemographic, health and lifestyle characteristics.

Age was a significant predictor of FIT uptake, with uptake pattern to FIT rising with age. This result coincides with other studies conducted in Spain and other countries (14, 28). Since CRC incidence increases with age, one possible explanation for this finding might be that people's risk perception about getting CRC increases with age, leading to a higher screening rate as they become older (29).

In terms of socioeconomic conditions, we discovered disparities based on educational level, social class and nationality. In our study, a higher educational level was a favorable predictor of uptake for FIT. Previously, a greater educational attainment has been linked to increased usage of preventive services, especially CRC screening (30). Compared to individuals with lower levels of education who may not perceive the value of screening, the higher educated group is related with improved risk perception, resulting in greater involvement in cancer screening (31). On the other hand, as in prior studies, the likelihood of complying with FIT improved with belonging to a higher social class (32). Belonging to a lower social class may explain poorer screening participation in terms of having low health literacy abilities, implying poor compliance with preventative health behaviors, and making cancer screening appear more dangerous, harder to complete, and less useful than it really is (33). Additionally, we found that FIT participation was markedly less common among immigrants than in Spanish people. According to the evidence, immigrant communities encounter a number of obstacles to health care access, including a lack of awareness of the Spanish National Health System and screening methods, as well as greater linguistic and cultural barriers (34). Considering these findings, attempts should be made to contact these populations, offering information in a variety of forms and languages, as well as providing translators, which may increase the engagement rate (35).

In the current study, FIT uptake varied according to the use of healthcare services. Specifically, participants who had more frequent contact with their General Practitioner were more likely to have undergone FIT. Health promotion is an important task in daily clinical practice, particularly among general practitioners, who are well-versed in cancer screening counseling (36). This could explain why people who have more contact with their primary care physician may receive recommendations on the importance of having a FIT.

Another notable result was that the number of reported chronic diseases was a favorable predictor of FIT uptake. This is consistent with previous research (37). While our findings are likely to be the result of more encounters with providers and therefore more opportunities to perform the suggested screening procedures, it is plausible that individuals with chronic illnesses may experience a significant treatment and self-management load, which may in turn lead to a refusal to participate in healthcare interventions which are not directly associated with their primary disorder (38). On the other hand, in the univariate analysis, mental health problems were associated with a higher FIT uptake. However, results from previous research findings are inconsistent, with some authors reporting no differences in CRC uptake between people with or without diagnosed mental illness (39), while others showed an inverse association (40). One possible explanation for our findings is that people with mental health issues visit primary care more frequently, giving them greater access to general practitioners' recommendations for cancer screening.

In our study, the decision to undergo FIT screening was also influenced by a variety of lifestyle factors. Thus, we found that consuming alcohol was positively associated with FIT uptake pattern, in line with past research (41). Although it is difficult to explain the positive relationship between alcohol intake and FIT uptake pattern, drinking alcohol, which is often linked with poor overall health (42), may lead people with poor overall health to be more inclined to request screening or follow-up after recommendations to undergo CRC screening from their family physician. On the other hand, physical activity has also been shown to be a positive factor with regard to uptake pattern to screening. Although Zamorano et al. (14) found no association, Thompson et al. (43) reported results that corroborated our findings, suggesting that physical activity was associated with increased participation rates. In this regard, it is believed that people who engage in healthy habits would be responsive to early screening procedures for disease control (44). Finally, findings from our study, as well as others (41, 45), have shown that current smoking is significantly related with a reduced likelihood of screening. The fact that current smokers have a decreased likelihood of cancer screening despite their greater CRC risk (46) is a worrying contradiction. Our study raises the hypothesis that reduced uptake pattern to FIT among current smokers may result in more advanced presentation and poorer outcomes.

This research offers an in-depth study of the factors influencing CRC screening uptake pattern. Even though CRC screening is a free population-based program in Spain, our results imply that socioeconomic inequalities in screening uptake pattern may exist. Our study is of use to the general public, healthcare professionals, researchers, and health policy makers, since a greater awareness of CRC screening discrepancies in Spain will benefit the general public. Among this population, it would be preferable if further reminders of the need to prevent illness, were sent by mail or by phone. Healthcare practitioners must be more aware of the low screening rates in Spain in order to identify screening barriers and continue to inform the public about the need for CRC screening. Those involved in research and health policy must design initiatives targeted at increasing CRC screening participation among populations with low screening uptake pattern.

The main strengths of our study lie in the fact that our study comprises a large representative sample of the Spanish population and that we can evaluate a wide variety of sociodemographic and health-related factors. However, certain limitations should also be noted. First, the results from the SNHS and EHSS may be influenced by non-response bias, recall bias or interviewers' proclivity to provide socially favorable replies. Second, the type of analytical method used (Guaiac or immunochemical fecal test) was not gathered in the SNHS or EHSS, despite the fact that FIT is now used in the majority of screening programs in Spain. Third, we were unable to discern whether FIT is due to screening or other reasons; as a result, the findings may have been overstated. Fourth, because the SNHS 2017 and the EHSS 2020 estimate the proportion of subjects up to date with FIT screening but do not report information about an invitation from the screening program, it is not possible to refer to adherence or participation in the FIT screening program. Fifth, it is not possible to determine whether FIT was performed on asymptomatic or symptomatic participants using data from the SNHS 2017 and EHSS 2020, as a result, the proportion of people who used the FIT as a screening method may be lower than indicated in the current manuscript. Finally, because a cross-sectional design was used, causation could not be deduced.

## 5. Conclusions

In Spain, although the evolution of FIT uptake pattern from 2017 to 2020 is positive, the prevalence of FIT uptake is still low (38.01%), not reaching the level considered as acceptable in the European guidelines. Factors such as being between 57 and 69 years, having a higher educational level or higher social class, having at least one chronic illness, being in frequent contact with the primary care physician, alcohol consumption and physical activity act as positive predictors to FIT uptake pattern, but immigration and smoking habit are negative predictors.

## Data availability statement

Publicly available datasets were analyzed in this study. This data can be found at: https://www.sanidad.gob.es/estadisticas/microdatos.do.

## Ethics statement

Ethical review and approval was not required for the study on human participants in accordance with the local legislation and institutional requirements. The patients/participants provided their written informed consent to participate in this study.

## Author contributions

SP: conceptualization, methodology, writing—original draft, resources, and data curation. SP and JC: data curation, data analysis, project administration, methodology, and writing—reviewing and editing. JC: visualization, data curation, and supervision. All authors contributed to the article and approved the submitted version.
